# Accuracy of Y-scope, a newly developed portable abdominal impedance analyzer, for the assessment of abdominal visceral fat area

**DOI:** 10.3389/fnut.2022.950747

**Published:** 2022-10-12

**Authors:** Ji Won Yoon, Minji Sohn, Ji Hye Moon, Soo Lim

**Affiliations:** ^1^Department of Internal Medicine, Healthcare System Gangnam Center, Seoul National University Hospital, Seoul, South Korea; ^2^Department of Internal Medicine, Seoul National University College of Medicine, Seoul, South Korea; ^3^Department of Internal Medicine, Seoul National University Bundang Hospital, Seongnam, South Korea

**Keywords:** bioimpedance analysis, abdominal impedance, body composition, visceral fat area, subcutaneous fat area

## Abstract

**Aim:**

This study was conducted to evaluate the accuracy of a newly developed multifrequency segmental (MFS) bioelectrical impedance analysis (BIA) method using an additional portable abdominal (PA) impedance analyzer, in the assessment of abdominal visceral fat area (VFA).

**Materials and methods:**

One hundred healthy Korean subjects aged 19 years or over (43 men and 57 women) were recruited, and VFA was estimated by a conventional MFS-BIA machine and a new MFS-BIA machine with a PA-BIA device, indicating MFS-VFA and MFS&PA-VFA, respectively. The accuracy of the VFA values was compared with those evaluated with CT at the level of the umbilicus (CT-VFA).

**Results:**

The mean age was 41 years and mean body mass index (BMI) was 24.4 kg/m^2^. The mean ± SD VFAs measured by CT, conventional MFS-BIA, and new MFS&PA-BIA together were 93.4 ± 60.9, 92.7 ± 53.4, and 93.6 ± 55.4 cm^2^, respectively. Correlation coefficients comparing CT-VFA with MFS-VFA and MFS&PA-VFA were 0.612 and 0.932, respectively (*P* < 0.001 for both). The mean difference between CT-VFA and MFS&PA-VFA was less affected by age, sex, and BMI compared with that between CT-VFA and MFS-VFA. Intraclass correlation coefficient (95% CI) between CT-VFA and MFS&PA-VFA was also greater than that between CT-VFA and MFS-VFA, 0.96 (0.95–0.98) vs. 0.76 (0.64–0.84), respectively.

**Conclusion:**

In this study, application of a newly developed MFS-BIA machine combined with a PA-BIA device significantly improved the correlation with CT-measured VFA without proportional error. This novel approach using advanced technology may be able to provide more reliable estimates of abdominal VFA.

## Introduction

Abdominal obesity is a condition in which excessive fat accumulates in the abdomen. It is a preventable disease that needs careful management because it is associated with a variety of other diseases, such as dyslipidemia, diabetes mellitus, atherosclerosis, and hypertension (1–3). During the COVID-19 pandemic, preventive procedures against the spread of COVID-19 have accelerated weight gain and given a negative influence on cardiometabolic profiles in subjects with metabolic impairments (4, 5).

Abdominal fat can be divided into subcutaneous fat and visceral fat according to its location. As the amount of visceral fat increases, the likelihood of cardiovascular and metabolic diseases such as hypertension and diabetes mellitus also increases (6, 7). For this reason, it is important to quantify abdominal fat accurately using a technology that is more appropriate for diagnosing abdominal obesity than waist circumference. Abdominal fat area or amount can be measured using CT and MRI. However, these methods have associated problems, such as high cost, low accessibility, and radiation exposure in the case of CT, and thus, their use in clinical practice is limited.

In contrast, the bioelectrical impedance analysis (BIA) method is easy to apply and is safe from a clinical perspective. BIA is a method of calculating body composition using the difference in electrical conductivity according to the biological characteristics of tissue (8). Recently, a technique that divides a human body into five virtual cylinders, i.e., four limbs and a trunk, and separately measuring the impedance of each cylinder has been widely used (9). While the muscles of the trunk occupy 50% of the entire body, impedance of the trunk is only one-tenth that of the other parts, so impedance changes only 1–2 Ω even when muscle mass changes significantly (10). For this reason, there is a limit to the utility of estimating trunk muscle mass or visceral fat area (VFA) using the impedance value of the trunk. Accordingly, much effort has been made to develop new devices with improved measurement accuracy. Of note, it has been shown that VFA measured by multifrequency segmental (MFS)-BIA has a significant correlation with that measured by reference methods, such as CT, MRI, or dual energy X-ray absorptiometry (DXA) (11–13). However, the correlation between MFS-BIA and CT in VFA measurement is modest and there is still some possibility for improvement. Also, in our previous study, we compared BIA and CT scan in measurements of VFA using an MFS-BIA machine (InBody720^®^; InBody Corporation, Seoul, South Korea) in men and women and found that there were some biases in the assessment of VFA by BIA compared with that by CT (14). Under this circumstance, a more sophisticated tool is needed to measure VFA precisely.

Conventional MFS-BIA measures axial impedance using electrodes that are in contact with the limbs. Since VFA is derived from information taken in cross section, it is expected that the estimated value of VFA obtained by CT can be improved by adding impedance also measured in cross section. Recently, a portable abdominal (PA) analyzer was developed for this purpose in 2020. This device measures waist circumference and abdominal transverse impedance, and therefore, allows estimation of VFA and subcutaneous fat area (SFA). In this study, we investigated how the estimated VFA value is improved compared to that from the existing MFS-BIA equipment by adding a portable abdominal BIA (PA-BIA) device.

## Materials and methods

### Study design and subjects

We recruited 100 healthy Korean subjects aged 20 years or over (43 men and 57 women) who agreed to participate in this study. Subjects who were contraindicated for CT scan or impedance analysis, such as pregnant women and those with a pacemaker, were excluded. Subjects who had a malignancy, stage 3–5 chronic kidney disease, liver cirrhosis, heart failure, or severe hypothyroidism were also excluded. The study protocol was approved by the ethics committee of Seoul National University Bundang Hospital (B-1711-432-003), and informed consent was obtained from all the study participants.

### Anthropometrics

All the measurements were performed from 10 to 12 a.m. after breakfast and before lunch, avoiding tests within 2 h after eating or 30 min after drinking water. Height and body weight were measured using standard protocols at the time of MFS-BIA. Body mass index (BMI) (kg/m^2^) was calculated by dividing body weight by height squared. Waist circumference was measured at the midpoint between the lateral iliac crest and the lowest rib at the end of expiration in the standing position with both feet about 25–30 cm apart. When the subcutaneous fat in the abdomen was excessive and overlapped at the measurement position, the subcutaneous fat was lifted, and measurements were taken.

### Visceral fat area estimation by multifrequency segmental bioelectrical impedance analysis

Abdominal VFA was estimated using an MFS-BIA machine (InBody770^®^, InBody Corporation, Seoul, South Korea), in a fasting state on the same day as the anthropometric measurements. The study participants were asked to refrain from smoking, drinking alcohol, and vigorous exercise for 48 h prior to measurement. After the subjects had been guided to stand on the foot electrode of the device, details of age and sex were entered into the machine. After confirming that the subject was standing correctly with both arms held at a 45° angle away from the body and that both feet were in the right place on the platform, assessment commenced. The device used 1, 5, 50, 250, 500 kHz, and 1 MHz frequencies to analyze body water content. MFS-VFA was calculated through a regression formula based on the relationship between the impedance value of the trunk and the visceral fat cross section taken by CT. The specific regression formula has not been published due to company confidentiality.

### Development of portable abdominal bioelectrical impedance analysis

Initially, the belt device was designed in consideration of the capability of measuring various body types as well as checking the waist circumference at the same time. This facility turned out to be disadvantageous in that it was difficult to place the electrode properly for subjects with a very large waist circumference and the measurer had to be in close contact with the subject.

To overcome this problem, a wheel device was developed for PA-BIA to measure body curvature and to easily adjust the angle of the electrode touching the body (Supplementary Figure 1). A PA-BIA using a Y-shaped electrode has the following advantages: (1) it can be measured in a standing position, as efficiently as other BIA measurements; (2) it can be applied in extreme cases with waist circumference less than 60 cm or greater than 110 cm because the angle of the electrode placement area can be adjusted; and (3) the entire electrodes are in direct contact with the skin of the abdomen without causing discomfort.

We believe that PA-BIA alone is not appropriate for examining body composition in the human body because it is calculated by combining the BMI, fat, and muscle distributions across the entire body in MFS-BIA.

### Visceral fat area and subcutaneous fat area estimation by multifrequency segmental portable abdominal bioelectrical impedance analysis

Measurement of abdominal impedance through the PA-BIA was performed at the flank of the right abdomen after wiping the skin with an electrolyte-soaked tissue or wet tissue. Sine waves of 50 and 250 kHz were generated through current electrodes at both ends of the PA-BIA, and as the current was passed through them, the voltage was measured through voltage electrodes inside the analyzer. The impedance (*Z* = V/I) was calculated from the fixed current and voltage values.

The InBody970^®^ instrument (InBody Corporation, Seoul, South Korea) is a newly developed device that uses frequencies of 1, 5, 50, 250, and 500 kHz, and 1, 2, and 3 MHz. The use of higher frequencies, such as 2 and 3 MHz, allows a constant current to flow over the body, thereby reflecting the intracellular and extracellular fluids more accurately. This minimizes the error of the InBody970^®^ in many cases. However, the accuracy of estimates of body composition measured by the InBody970^®^ instrument has been reported to be non-significantly higher than those measured by the InBody770^®^ instrument (15, 16).

Waist circumference was calculated by measuring the right half of the subject’s waist circumference by PA-BIA and multiplying it by two. Standing on the right side of the subject, the left side of the instrument was placed on the umbilicus and turned half a turn horizontally toward the spine.

Visceral fat area and SFA were estimated using both axial and transverse impedance values measured by a new MFS-BIA (InBody970^®^) machine and a PA-BIA device (Y-scope^®^ ; InBody Corporation, Seoul, South Korea), respectively. Waist circumference measured by the PA-BIA device was also used for estimating VFA and SFA. VFA, SFA, and waist circumference were measured six times, and the averages of these measurements were used in the analysis.

### Visceral fat area and subcutaneous fat area measurement using computed tomography scan

Abdominal adipose tissue areas were quantified by CT scan at a 90 kV exposure (Somatom Sensation 16; Siemens, Munich, Germany). A 10 mm CT slice scan was acquired at the umbilical level to measure visceral fat and SFA by measuring the mean value of all the pixels within the range of –190 to –50 Hounsfield units.

### Statistical analyses

The number of samples was determined based on a previous study that investigated differences in VFA measured with CT and conventional MFS-BIA (14). In this study, the mean ± SD of CT-VFA was 131.9 ± 57.3 cm^2^ in participants whose body size was similar to that in our current study population. Therefore, we hypothesized that we would find a clinically meaningful significant difference in fat measurements between conventional MFS-BIA and the new MFS-BIA used with the PA-BIA when the difference reached 10 cm^2^. Based on this assumption, we calculated a sample size of at least 90 people, and we chose a sample of 100 people for the current study.

Data are presented as the mean and SD and were analyzed using SPSS Windows version 25.0 (IBM Corporation, Armonk, NY, USA). Clinical characteristics by sex were compared using Student’s *t*-tests. One-way ANOVA was used to evaluate the difference according to age and BMI. Pearson’s correlation analyses were used to investigate any concordance between measurements by two different methods. To assess the significance of the difference between two correlation coefficients, Fisher’s *r*-to-*z* transformation was used. The comparability and agreement levels between two methods were evaluated using the Bland–Altman method. The paired *t*-test and intraclass correlation coefficient (ICC) for absolute agreement were used to assess the consistency or conformity of measurements made by two different methods. *P* < 0.05 was considered statistically significant.

## Results

### Characteristics of study participants

Table 1 summarizes the anthropometric characteristics of the study population (*n* = 100). Mean age was 41 years old and mean BMI was 24.4 kg/m^2^. Waist circumferences (mean ± SD) by direct measurement and PA-BIA were 85.5 ± 15.1 and 87.1 ± 15.4 cm, respectively.

**TABLE 1:** Clinical characteristics of study subjects.

Variables	Total (*n* = 100)	Men (*n* = 43)	Women (*n* = 57)	*P* ^a^
Age (years)	41.0 ± 16.5	38.5 ± 15.9	43.0 ± 16.8	0.178
Height (cm)	166.3 ± 10.8	175.0 ± 8.7	159.8 ± 7.0	< 0.001
Weight (kg)	68.4 ± 21.2	81.9 ± 22.4	58.2 ± 13.3	< 0.001
Body mass index (kg/m^2^)	24.4 ± 5.7	26.5 ± 6.0	22.8 ± 5.1	0.001
Waist circumference (cm)	85.5 ± 15.1	92.1 ± 15.4	80.4 ± 12.8	< 0.001
**Computed tomography**				
Visceral fat area (cm^2^)	93.4 ± 60.9	122.6 ± 59.0	71.4 ± 52.9	< 0.001
Subcutaneous fat area (cm^2^)	207.9 ± 135.5	225.4 ± 162.4	194.7 ± 110.7	0.264
**MFS-BIA (InBody770^®^)**				
Visceral fat area (cm^2^)	92.7 ± 53.4	96.4 ± 58.0	90.0 ± 50.1	0.554
**MFS&PA-BIA (InBody970^®^ + Y-scope^®^)**				
Waist circumference (cm)	87.1 ± 15.4	93.8 ± 15.7	82.1 ± 13.3	< 0.001
Visceral fat area (cm^2^)	93.6 ± 55.4	118.2 ± 53.4	75.1 ± 49.6	< 0.001
Subcutaneous fat area (cm^2^)	209.6 ± 132.8	228.9 ± 153.0	195.0 ± 114.7	0.208

Values are expressed as group mean ± SD. MFS, multifrequency segmental; BIA, bioimpedance analysis; PA, portable abdominal.

^a^*P*-value by Student’s *t*-test between men and women.

Visceral fat area (mean ± SD) by CT (CT-VFA), a conventional MFS-BIA alone (MFS-VFA), and a new MFS-BIA and PA-BIA together (MFS&PA-VFA) were 93.4 ± 60.9, 92.7 ± 53.4, and 93.6 ± 55.4 cm^2^, respectively. SFA (mean ± SD) by CT (CT-SFA) and MFS-BIA and PA-BIA together (MFS&PA-SFA) were 207.9 ± 135.5 and 209.6 ± 132.8 cm^2^, respectively.

Body mass index and waist circumference were both significantly greater in men than women (*P* < 0.01). CT-VFA and MFS&PA-VFA were significantly greater in men than women (*P* < 0.01). SFA measured by CT and estimated by MFS-BIA and PA-BIA together did not differ according to sex (*P* = 0.264 and *P* = 0.208, respectively).

### Comparison between visceral fat areas measured by computed tomography and multifrequency segmental bioelectrical impedance analysis methods

**FIGURE 1:**
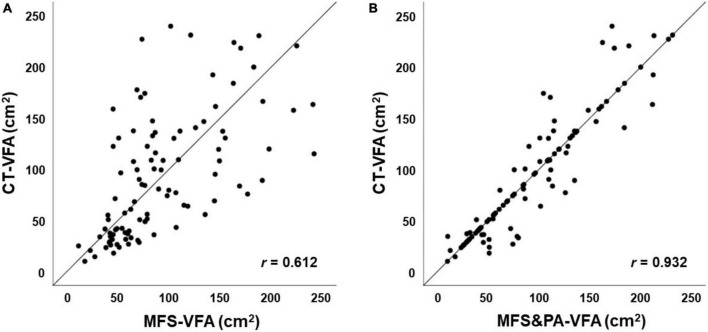
Correlation between VFA by different methods. **(A)** MFS-VFA and CT-VFA; **(B)** MFS&PA-VFA and CT-VFA. CT, computed tomography; VFA, visceral fat area; CT-VFA, VFA measured by CT; MFS-VFA, VFA measure by a multifrequency segmental bioelectrical impedance analysis (BIA) machine (InBody770^®^); MFS&PA-VFA, VFA measured by a new MFS-BIA machine combined with a portable abdominal BIA device (InBody970^®^ + Y-scope^®^).

In the case of VFA, the correlation coefficient between CT-VFA and MFS-VFA was 0.612 (*P* < 0.001, Figure 1). The Bland–Altman plot showed that the mean difference between CT-VFA and MFS-VFA was close to zero (0.7 ± 50.8 cm^2^, *P* for paired *t*-test = 0.889). However, the 95% CI is as wide as 100 cm^2^ and there is a tendency for the difference to increase as the mean of the two values increases.

**FIGURE 2:**
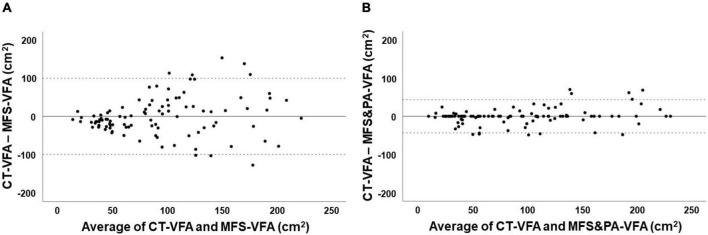
Bland–Altman plot for comparing the two methods. **(A)** MFS-VFA and CT-VFA; **(B)** MFS&PA-VFA and CT-VFA. CT, computed tomography; VFA, visceral fat area; CT-VFA, VFA measured by CT; MFS-VFA, VFA measure by a multifrequency segmental bioelectrical impedance analysis (BIA) machine (InBody770^®^); MFS&PA-VFA, VFA measured by a new MFS-BIA machine combined with a portable abdominal BIA device (InBody970^®^ + Y-scope^®^).

The mean difference between CT-VFA and MFS&PA-VFA was nearly zero (–0.2 ± 22.2 cm^2^), and the 95% CI (–43.6 to 43.3 cm^2^, *P* for paired *t*-test = 0.943) was smaller than that between CT-VFA and MFS-VFA. No apparent trend was observed in the difference between CT-VFA and MFS&PA-VFA throughout the average values of the two methods (Figure 2 and Table 2).

**TABLE 2:** Subgroup analyses of mean differences for visceral fat area.

	*N*	CT-VFA (cm^2^)	MFS-VFA (cm^2^)	MFS&PA-VFA (cm^2^)	Difference in VFA (cm^2^): CT—MFS	Difference in VFA (cm^2^): CT—MFS&PA^b^	P1^c^	P2^d^	ICC1^e^	ICC2^f^
Total	100	93.4 ± 60.9	92.7 ± 53.4	93.6 ± 55.4	0.7 ± 50.8	–0.2 ± 22.2			0.76 (0.64–0.84)	0.96 (0.94–0.98)
**Sex**										
Men	43	122.6 ± 59.0	96.4 ± 58.0	118.2 ± 53.4	26.2 ± 54.5^a^	4.5 ± 25.3	<0.001	0.068	0.68 (0.36–0.84)	0.95 (0.90–0.97)
Women	57	71.4 ± 52.9	90.0 ± 50.1	75.1 ± 49.6	–18.5 ± 38.2^a^	–3.7 ± 19.0			0.81 (0.63–0.90)	0.96 (0.94–0.98)
**Age (years)**										
20–29	37	71.3 ± 50.5	97.0 ± 65.5	74.6 ± 53.3	–25.7 ± 39.3^a^	–3.3 ± 16.6	<0.001	0.465	0.83 (0.52–0.93)	0.97 (0.95–0.99)
30–39	14	103.7 ± 57.2	110.8 ± 53.0	106.7 ± 52.0	–7.2 ± 31.1	–3.1 ± 19.8			0.92 (0.74–0.97)	0.97 (0.90–0.99)
40–59	39	98.6 ± 62.4	81.1 ± 41.7	96.8 ± 54.2	17.5 ± 52.9^a^	1.8 ± 21.1			0.65 (0.34–0.82)	0.97 (0.94–0.98)
≥60	10	140.8 ± 68.2	96.9 ± 40.4	132.9 ± 50.2	44.0 ± 52.3^a^	7.9 ± 41.4			0.62 (0.33–0.90)	0.87 (0.49–0.97)
**Body mass index (kg/m^2^)**										
<21.3	34	50.1 ± 41.0	51.8 ± 16.6	51.6 ± 38.1	–1.7 ± 37.9	–1.5 ± 14.9	0.279	0.908	0.43 (–0.17 to 0.72)	0.96 (0.93–0.98)
21.3–25.2	33	87.6 ± 48.6	75.8 ± 24.4	86.8 ± 33.9	11.8 ± 45.1	0.8 ± 28.2			0.47 (–0.05 to 0.73)	0.88 (0.75–0.94)
≥25.2	33	144.0 ± 52.1	151.8 ± 47.7	143.7 ± 48.2	–7.9 ± 65.3	0.3 ± 22.2			0.26 (–0.52 to 0.64)	0.95 (0.90–0.98)

CT, computed tomography; VFA, visceral fat area; CT-VFA, VFA measured by CT; MFS-VFA, VFA measure by a multifrequency segmental bioelectrical impedance analysis (BIA) machine (InBody770^®^); MFS&PA-VFA, VFA measured by a new MFS-BIA machine combined with a portable abdominal BIA device (InBody970^®^ + Y-scope^®^); CT-MFS, differences between VFAs measured by CT and MFS-BIA; CT-MFS&PA, differences between VFAs measured by CT and MFS&PA-BIA; intraclass correlation coefficient.

^a^*P* < 0.05, paired *t*-test between CT-VFA and MFS-VFA.

^b^No difference shown between CT-VFA and MFS&PA-VFA by paired *t*-test.

^c^P1, *t*-test or ANOVA among subgroups, CT-MFS.

^d^*P2*, *t*-test or ANOVA among subgroups, CT- MFS&PA.

^e^ICC1, intraclass correlation coefficient between CT-VFA and MFS-VFA.

^f^ICC2, intraclass correlation coefficient between CT-VFA and MFS&PA-VFA.

Overall, the difference between CT-VFA and conventional MFS-VFA was not significant (*P* = 0.889). However, on subgroup analysis, the difference was significant in both sexes (26.2 ± 54.5 cm^2^, *P* = 0.003 in men, –18.5 ± 38.2 cm^2^, *P* = 0.001 in women), indicating an underestimation in men and an overestimation in women by MFS-BIA. Furthermore, subjects aged under 30 years or over 39 years showed a significant difference between CT-VFA and MFS-VFA (*P* < 0.05, respectively). The mean difference between CT-VFA and MFS-VFA was significantly different according to sex and age (*P* < 0.001 for ANOVA). The difference between CT-VFA and MFS-VFA was not significant across the BMI tertile groups.

On the other hand, the correlation coefficient between CT-VFA and MFS&PA-VFA was 0.932 (*P* < 0.001). When correlations were compared using Fisher’s r-to-z transformation, MFS&PA-VFA indicated greater correlation with CT-VFA than MFS-VFA (*z* = 6.69, *P* < 0.001). There was no difference between CT-VFA and MFS&PA-VFA not only in the total population, but also in sex, age, and BMI subgroups (*P* > 0.05).

**FIGURE 3:**
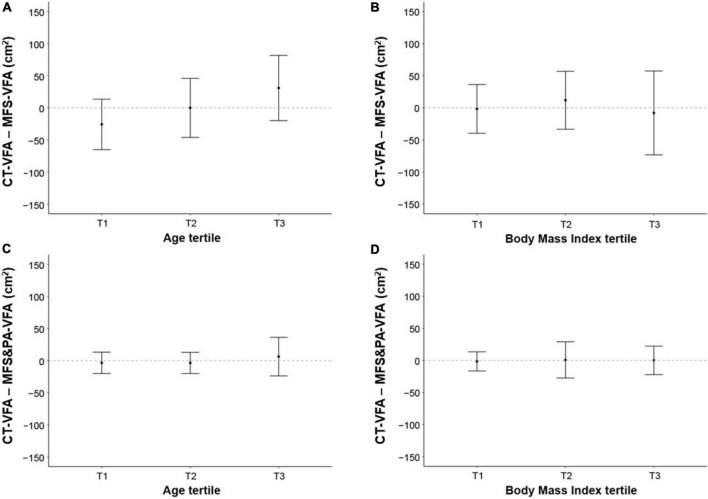
Differences in VFAs between two methods according to **(A,C)** age and **(B,D)** body mass index subgroups. Data are indicated as mean ± SD. CT, computed tomography; VFA, visceral fat area; CT-VFA, VFA measured by CT; MFS-VFA, VFA measure by a multifrequency segmental bioelectrical impedance analysis (BIA) (InBody770^®^); MFS&PA-VFA, VFA measured by a new MFS-BIA machine combined with a portable abdominal BIA device (InBody970^®^ + Y-scope^®^).

The ICCs (95% CI) for MFS-VFA and MFS&PA-VFA compared with CT-VFA were 0.76 (0.64–0.84) and 0.96 (0.94–0.98), respectively. Given that the 95% CIs did not overlap, the ICC between CT-VFA and MFS&PA-VFA was significantly higher than that between CT-VFA and MFS-VFA (Table 2). Among subgroups according to sex and age, MFS-VFA values were different from CT-VFA values, while MFS&PA-VFA were not (Table 2). No difference was found among BMI subgroups, while the difference subtracted to CT-VFA was smaller with MFS&PA-VFA than MFS-VFA (Figure 3).

### Comparison between subcutaneous fat areas measured by computed tomography and multifrequency segmental portable abdominal bioelectrical impedance analysis

The correlation coefficient between CT-SFA and MFS&PA-SFA was 0.984 (*P* < 0.001) (Supplementary Figure 2). The mean difference between CT-SFA and MFS&PA-SFA was –1.6 ± 24.4 cm^2^ (*P* for paired *t*-test = 0.509). No apparent trend was observed in the difference between CT-SFA and MFS&PA-SFA throughout the average values of the two methods (Supplementary Figure 3).

No difference was found between CT-SFA and MFS&PA-SFA among subgroups according to sex, age, and BMI (*P* > 0.05, Supplementary Table 1). ICCs were consistently high (>0.9) with low differences in every subgroup (Supplementary Table 1 and Supplementary Figure 4).

## Discussion

From our study performed in a healthy Korean population, VFA measured by MFS-BIA and PA-BIA together was more correspondent than that measured by CT-VFA without significant proportional error than MFS-BIA alone. This was confirmed by ICC analysis, suggesting that MFS&PA-VFA was superior to MFS-VFA in terms of absolute agreement with CT-VFA. CT-VFA and MFS&PA-VFA were not different not only in the total population, but also in the subgroups of sex, age, and BMI.

Visceral fat is regarded as playing a key role in the pathogenesis of insulin resistance, chronic inflammation (17, 18), and type 2 diabetes (19). In addition, visceral obesity is included as the most important factor among the diagnostic criteria for metabolic syndrome (20). It also plays an important role in the development of atherosclerosis (21). Herein, it lies the importance of an accurate assessment of the amount of visceral fat.

Among several methods of measuring VFA, CT scan is considered the gold standard (22). However, BIA has several advantages compared to CT. BIA is a non-invasive and low-cost test with no radiation exposure. It requires less scan time and is easy and safe to measure, and is, thus, suitable not only for daily clinical practice, but also for epidemiological studies on a large scale (23).

Since abdominal impedance is only about 5–10% of that of the limbs, measuring visceral fat by conventional BIA is not as accurate as for whole-body fat (23). Currently, however, MFS-BIA is widely used with improved accuracy in estimating VFA (12, 14, 24, 25). In a study comparing patients with liver disease in Japan, the correlation coefficient of VFA measured by two methods was 0.7. Another study of 102 men and women showed a correlation coefficient of 0.64 (25, 26). In several other studies, VFA measured by MFS-BIA correlated significantly with VFA measured by CT, but there was a difference in concordance rate according to age and sex (14, 24).

In our study, in line with previous studies, MFS-VFA was significantly correlated with CT-VFA, although the correlation was not robust (*r* = 0.612) and systematic error was suggested by the difference increasing as the mean of the two values increased. Also, underestimation in men and overestimation in women were observed in MFS-BIA of VFA. In addition, the difference between CT-VFA and MFS-VFA was significant at both extremes of age. Regarding BMI, no significant difference was observed between CT-VFA and MFS-VFA in each subgroup. However, the overall difference was larger than when compared with MFS&PA-VFA.

To overcome the limitations of conventional BIA, Scharfetter et al. (27) developed a new technique for quantifying abdominal subcutaneous fat thickness with electrical impedance across the waist. Subsequently, Ryo et al. (28) reported measurement of the VFA using local BIA. In this study, correlation with VFA determined by CT was significant (*r* = 0.88) (28). In another study, the correlation coefficients between abdominal BIA and MRI in the assessment of visceral fat were 0.65 in men and 0.64 in women (29). In these studies, only abdominal BIA measuring transverse impedance was used for VFA estimation. In the study using dual BIA to measure transverse and axial impedance, higher accuracy and better correlation with metabolic indices were observed with dual BIA as compared with conventional MFS-BIA (26).

In our study, the VFA value was calculated by considering impedance values measured both by conventional MFS-BIA and PA-BIA. As a result, VFA values derived in this way showed significantly higher correlation and agreement with measured CT values. Unlike conventional MFS-BIA, no difference in accuracy according to sex, age, and BMI was observed in VFA estimation when PI-BIA was added. Absolute agreement in the assessment of VFA between CT and MFS-BIA combined with PA-BIA was excellent (95% CI of ICC = 0.94–0.98).

By using PA-BIA, it was possible to obtain SFA values that could not be obtained from conventional MFS-BIA. In our study, MFS&PA-SFA showed excellent correlation and agreement with CT-SFA without systematic error.

The strength of our study lies in the comparative evaluation of the accuracy of conventional BIA, which is currently widely used for VFA measurement, and the new method incorporating the addition of PA-BIA, with the gold standard, CT. Moreover, agreement as well as correlation could be evaluated through various statistical analyses. Furthermore, by including broad-spectrum BMI groups including subjects with underweight and with morbid obesity and subjects of various age groups, the effect of measurement accuracy by sex, age, and BMI could be analyzed.

There were also some limitations in our study. We recruited only Koreans that may make it difficult to apply the current results to other populations. We did not measure various biochemical parameters and the metabolic status of each individual might affect the BIA measures.

## Conclusion

In conclusion, the abdominal BIA method offers a useful instrument that can be applied in routine clinical practice for the evaluation of VFA, a condition that is significantly associated with cardiometabolic impairment. In our study, adding additional information obtained from a specialized device on abdominal impedance was shown to be highly correlated and in increased agreement with CT-measured VFA without proportional error. Thus, application of PA-BIA to MFS-BIA significantly improves the accuracy of abdominal VFA measurements. This novel advanced technology may be able to provide a more reliable estimate of abdominal VFA.

## Data availability statement

The raw data supporting the conclusions of this article will be made available by the authors, without undue reservation.

## Ethics statement

The studies involving human participants were reviewed and approved by Institutional Review Board of Seoul National University Bundang Hospital. The patients/participants provided their written informed consent to participate in this study.

## Author contributions

SL conceptualized the project. JY and SL designed the study, analyzed the data, and wrote the manuscript. JM performed the experiments. MS provided technical and/or conceptual assistance. All authors read and approved the manuscript.
