# Supplementary material for: Accuracy of Y-scope, a newly developed portable abdominal impedance analyzer, for the assessment of abdominal visceral fat area
Source: Front Nutr. 2022 Oct 12;9:950747. doi: 10.3389/fnut.2022.950747 (PMC9597369; doi:10.3389/fnut.2022.950747)
Supplement: Supplementary file 1 [file Table_1.DOCX]

| **Supplementary Table 1. Subgroup analyses of mean differences for subcutaneous fat area** | | | | | | | | | | | | |
| --- | --- | --- | --- | --- | --- | --- | --- | --- | --- | --- | --- | --- |
|  | N | CT-SFA (cm^2^) | | | MFS&PA-SFA (cm^2^) | | | Difference in SFA (cm^2^)  (CT – MFS&PA)*^a^* | | | *P^b^* | ICC^c^ |
| Total | 100 | 207.9 | ± | 135.5 | 209.6 | ± | 132.8 | –1.6 | ± | 24.4 |  | 0.99 (0.99–0.99) |
| *Sex* | | | | | | | | | | | | |
| Men | 43 | 225.4 | ± | 162.4 | 228.9 | ± | 153.0 | –3.4 | ± | 24.5 | 0.521 | 0.99 (0.99–1.00) |
| Women | 57 | 194.7 | ± | 110.7 | 195.0 | ± | 114.7 | –0.2 | ± | 24.5 |  | 0.99 (0.98–0.99) |
| *Age (years)* | | | | | | | | | | | | |
| 20–29 | 37 | 237.4 | ± | 178.1 | 237.4 | ± | 169.8 | 0.1 | ± | 23.1 | 0.758 | 0.99 (0.99–1.00) |
| 30–39 | 14 | 245.4 | ± | 140.6 | 253.6 | ± | 133.8 | –8.1 | ± | 20.9 |  | 0.99 (0.98–1.00) |
| 40–59 | 39 | 177.1 | ± | 83.1 | 177.9 | ± | 90.0 | –0.9 | ± | 25.4 |  | 0.98 (0.96–0.99) |
| ≥ 60 | 10 | 166.3 | ± | 73.6 | 168.4 | ± | 79.6 | –1.8 | ± | 31.8 |  | 0.96 (0.83–0.99) |
| *Body mass index (kg/m^2^)* | | | | | | | | | | | | |
| < 21.3 | 34 | 112.5 | ± | 45.2 | 110.5 | ± | 46.4 | 2.0 | ± | 16.3 | 0.561 | 0.97 (0.94–0.98) |
| 21.3–25.2 | 33 | 168.1 | ± | 63.0 | 171.0 | ± | 57.6 | –2.9 | ± | 21.9 |  | 0.97 (0.93–0.98) |
| ≥ 25.2 | 33 | 346.2 | ± | 140.2 | 350.2 | ± | 129.2 | –4.0 | ± | 32.8 |  | 0.99 (0.97–0.99) |
| CT, computed tomography; VFA, visceral fat area; CT-VFA, VFA measured by CT; MFS-VFA, VFA measure by a multifrequency segmental bioelectrical impedance analysis (BIA) machine (InBody770^®^); MFS&PA-VFA, VFA measured by a new MFS-BIA machine combined with a portable abdominal BIA device (InBody970^®^+Y-scope^®^).  ^a^No difference shown between CT-SFA and MFS&PA-SFA by paired *t*-test. ^b^*P* value of *t*-test or ANOVA among subgroups, CT-SFA and MFS&PA-SFA. ^c^Intraclass correlation coefficient (ICC), between CT-SFA and MFS&PA-SFA | | | | | | | | | | | | |

**Supplementary Fig. 1. A new multifrequency segmental BIA and portable abdominal BIA.** **(A)** Multifrequency segmental bioelectric impedance analysis (BIA) (InBody970^®^); **(B)** Measurement of transverse abdominal impedance by a portable abdominal BIA (Y-scope^®^); **(C)** Front and side images (left) and functions of each part (middle and right) of the portable abdominal BIA.

**
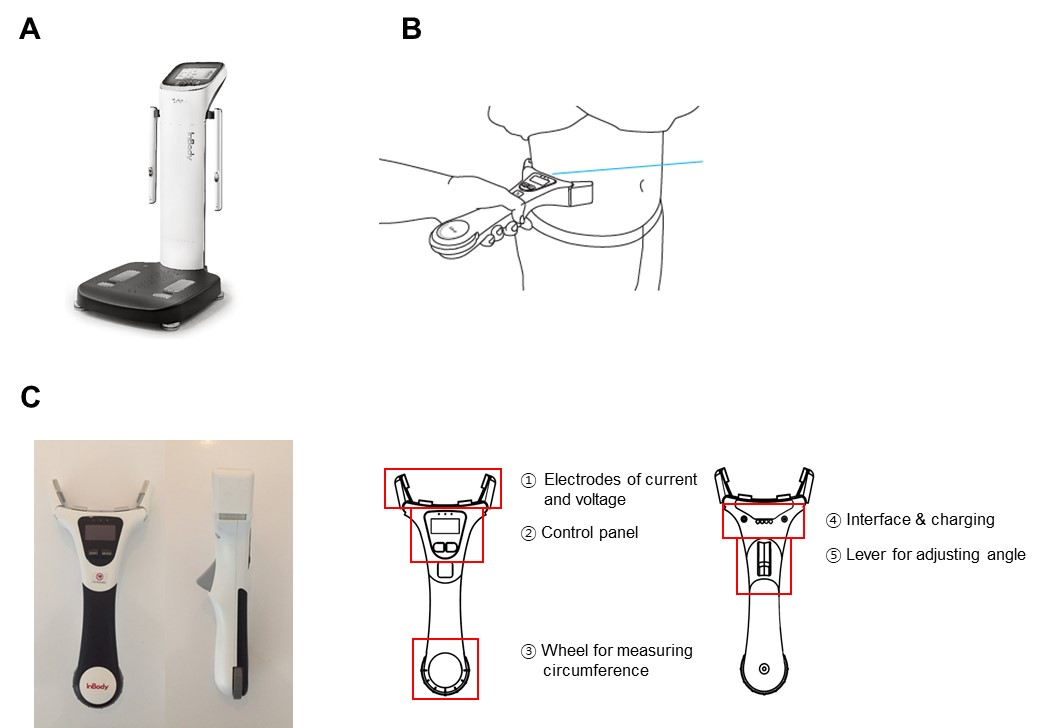
**

**Supplementary Fig. 2. Correlation between SFA by CT and MFS&PA-BIA (InBody970**^®^**+Y-scope**^®^**).** SFA, subcutaneous fat area; CT, computed tomography; **MFS&PA-BIA**; multifrequency segmental bioelectrical impedance analysis (BIA) combined with a portable abdominal BIA. CT-SFA, SFA measured by CT; MFS&PA-SFA, SFA measured by a new MFS-BIA machine combined with a portable abdominal BIA device.


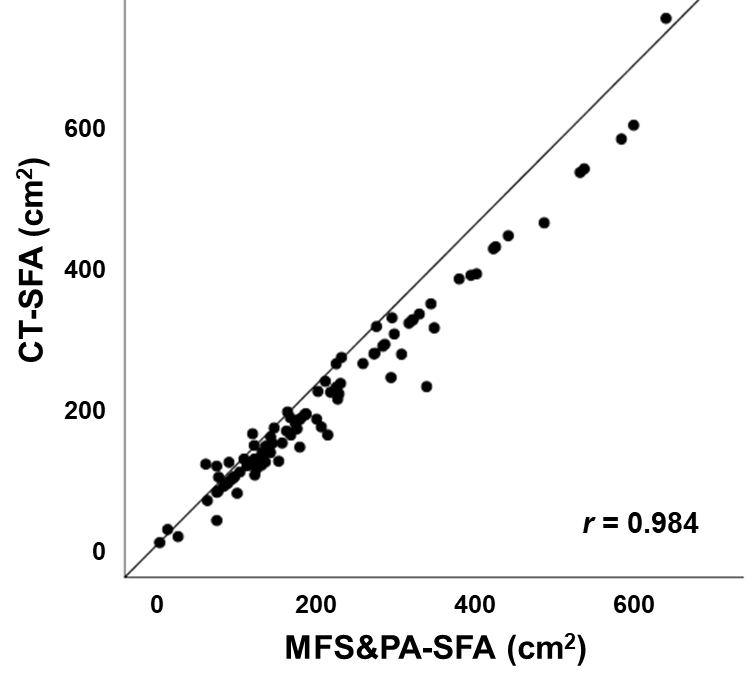


**Supplementary Fig. 3. Bland–Altman plot for comparing the two methods.** SFA, subcutaneous fat area; CT, computed tomography; MFS&PA-BIA; multifrequency segmental bioelectrical impedance analysis (BIA) combined with portable abdominal BIA. CT-SFA, SFA measured by CT; MFS&PA-SFA, SFA measured by a new MFS-BIA machine combined with a portable abdominal BIA device (InBody970^®^+Y-scope^®^).


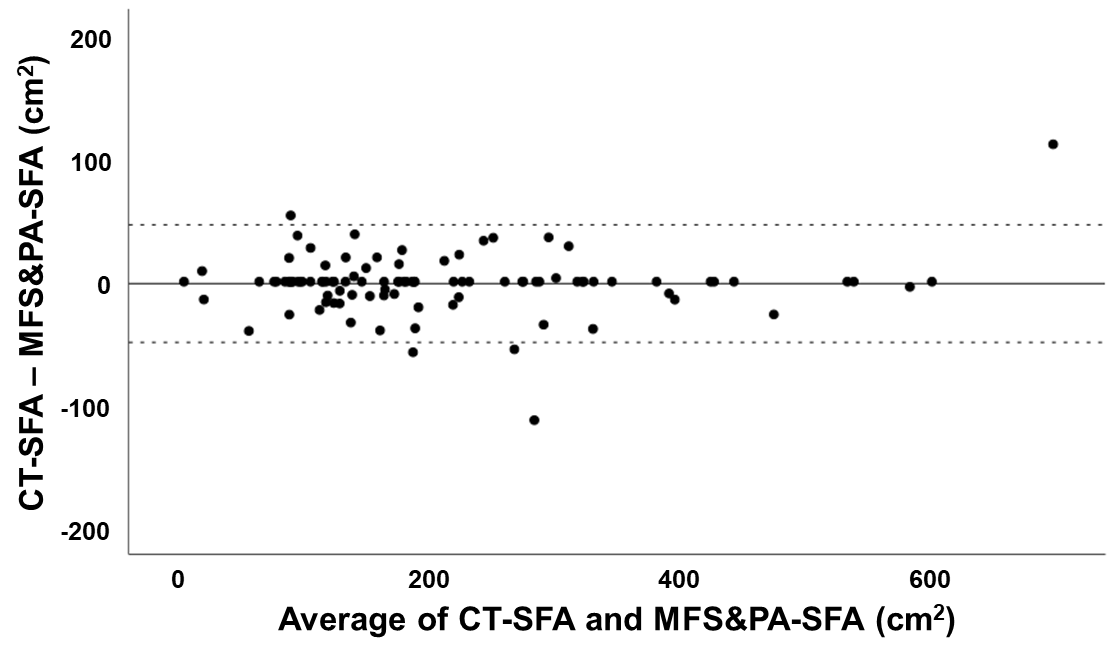


**Supplementary Fig. 4. Differences in SFAs between two methods according to (A) age and (B) body mass index subgroups.** SFA, subcutaneous fat area; CT, computed tomography; CT-SFA, SFA measured by CT; MFS&PA-SFA, SFA measured by a multifrequency segmental bioelectrical impedance analysis (BIA) machine combined with a portable abdominal BIA device (InBody970^®^+Y-scope^®^).

**
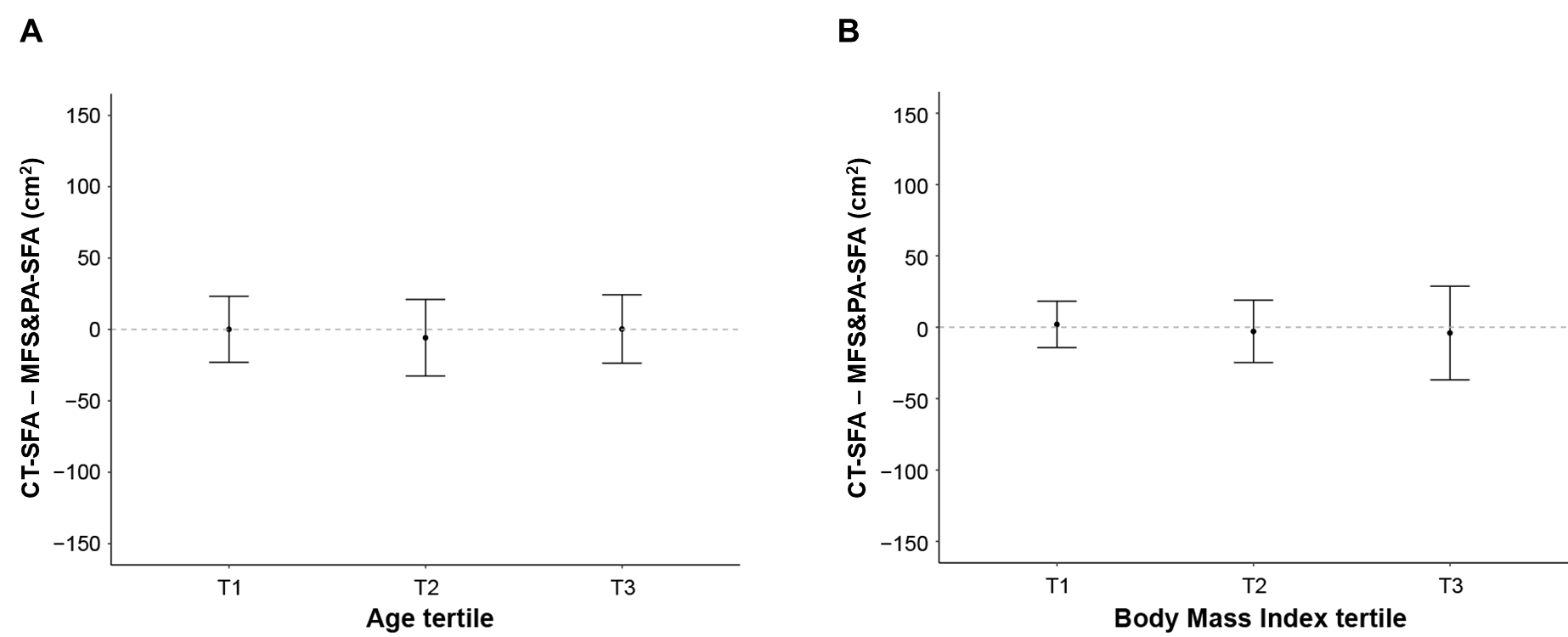
**
